# Bullying and Cyberbullying: Their Legal Status and Use in Psychological Assessment

**DOI:** 10.3390/ijerph14121449

**Published:** 2017-11-24

**Authors:** Muthanna Samara, Vicky Burbidge, Aiman El Asam, Mairéad Foody, Peter K. Smith, Hisham Morsi

**Affiliations:** 1 Department of Psychology, Kingston University, Penrhyn Road, Kingston upon Thames, London KT1 2EE, UK; vickyburbidge@hotmail.com (V.B.); A.ElAsam@kingston.ac.uk (A.E.A.); 2 Anti Bullying Research and Resource Centre, Dublin City University, Dublin D09 AW21, Ireland; mairead.foody@dcu.ie; 3 Department of Psychology, Goldsmiths College, University of London, London SE14 6NW, UK; P.Smith@gold.ac.uk; 4 National Centre for Cancer Care and Research (NCCCR), Hamad Medical Corporation (HMC), Doha 1705, Qatar; HMorsi1@hamad.qa

**Keywords:** bullying, cyberbullying, psychologist, psychiatrist, mental health, psychological assessment, psychological service, psychopathology, lawyer, legal

## Abstract

Bullying and cyberbullying have severe psychological and legal consequences for those involved. However, it is unclear how or even if previous experience of bullying and cyberbullying is considered in mental health assessments. Furthermore, the relevance and effectiveness of current legal solutions has been debated extensively, resulting in a desire for a specific legislation. The purpose of this study is to investigate the psychological and legal components of bullying and cyberbullying. This is a qualitative research that includes interviews with five practitioner psychologists and four lawyers in the United Kingdom (UK). Thematic analysis revealed three main themes. One theme is related to the definition, characteristics, and impact of bullying and cyberbullying and the need for more discussion among the psychological and legal professions. Another theme is related to current professional procedures and the inclusion of questions about bullying and cyberbullying in psychological risk assessments. The third theme emphasised the importance of intervention through education. Two key messages were highlighted by the lawyers: ample yet problematic legislation exists, and knowledge will ensure legal success. The study recommends the necessity of performing revisions in the clinical psychological practices and assessments, and the legal policies regarding bullying and cyberbullying. In addition to improving legal success, this will reduce bullying prevalence rates, psychological distress, and psychopathology that can be comorbid or emerge as a result of this behaviour.

## 1. Introduction

Bullying is generally regarded as an intentional, repeated, aggressive act that is carried out over time, with a power imbalance between the bully and the victim [1]. Cyberbullying adheres to the same definition but with the use of the internet and/or electronic devices [2]. The anonymity of the bully and their skilled use of technology can cause the power imbalance [3]; while repetition arises because different people can continually view the act over a short period of time [4]. Cyberbullying takes multiple forms, such as flaming, harassment, defamation, denigration, impersonation, outing, exclusion, and cyberstalking [5,6,7].

Bullying (throughout the remainder of the paper, the term “bullying” will be used to cover both traditional and cyber forms unless it is explicitly stated otherwise), whether traditional or cyberbullying [8], has psychological consequences for everyone involved, not just victims [9,10]. Wellbeing is influenced by type, frequency, and duration of bullying [11]. Being a bully is a risk factor for depression, anxiety, eating disorders, and substance abuse [12,13], whilst victims report high rates of self-injury and suicidal ideation [8]. Victims can experience long term health risks, continuing into adulthood [14], even forty years after the experience has passed [15]. Cyber victims have also been shown to have low self-esteem, high levels of depressive symptoms [16], and emotional and peer problems [17], whereas cyber bullies have demonstrated conduct problems, hyperactivity, and low pro-social behaviour [17]. Bully/victims are most vulnerable in terms of psychopathology, experiencing some of the associated risks and sharing the characteristics of both perpetrators and victims [13,14,17,18]. The stronger influence of child inherent characteristics makes it likely that rather than being passive victims, they turn to become bullies as well. For example, a child who is bullied by someone stronger may react by bullying someone weaker to regain their status and/or self-esteem [19]. Involvement in traditional and cyber bullying has also been related to the increased use of alcohol and drugs [20,21,22,23,24,25,26,27], which is a relevant risk factor that is associated with suicidal attempts and behaviors [28]. There are thus serious psychological risks for all involved, and the physical health of victims may be compromised due to stress [29].

Because of the above mentioned consequences, preventing bullying can reduce self-harming behaviours [30]. Idsoe et al. [31] argue that if children report being bullied they should be assessed for trauma related symptoms. Interventions are crucial and “early detection is central to the prevention of [the] long-term effects” [32] (p. 39). Because of the longevity of its impact, Sourander et al. [17] argue that questions about previous bullying experiences should be included in consultations with mental health practitioners. However, it is not known how many people consult a practitioner, or, if they do, whether practitioners actively consider bullying in their consultations.

It is assumed that because of the strong psychological risks, those involved in bullying are more likely to seek psychological help [33]. In the United Kingdom (UK), General Practitioners (GPs), as a first point of contact, should be able to recognise verbal and non-verbal cues about bullying [29]. The literature (see [29,34,35,36]) highlights ways in which GPs can do so, however guidelines about bullying for practitioner psychologists are limited. We would argue that all healthcare practitioners should have relevant guidelines to bullying, and the void recognised by Dale et al. [33] between research and its application in healthcare systems must be rectified.

To diagnose mental health issues, and to provide a common language for communicating diagnoses, psychologists use diagnostic manuals, such as the Diagnostic and Statistical Manual and Mental Disorders (DSM) and the International Classification of Diseases (ICD). However, bullying is mentioned only once, as a criterion of conduct disorder, within DSM [37], and only one broad classification exists in the ICD-10 [38]. It is referred to in five of the National Institute for Health and Care Excellence (NICE) guidelines (Social anxiety disorder (CG159), social and emotional wellbeing (LGB12), obesity in children and young people (PH47), bedwetting in adolescence (CG111), and depression in children and young people (CG28)), suggesting that bullying should be within the practitioner psychologist’s assessment. The guidelines for depression in children and young people (CG28) refer extensively to bullying as a risk factor of depression, highlighting that the training of healthcare practitioners is imperative in recognising the symptoms of bullying. However, the overall reference to bullying in these guidelines, and in psychological practice, might be limited and we do not know to what extent, if at all, practitioner psychologists are screening for bullying.

The literature highlights the need for further research to explore how bullying is integrated into client consultations. One of the objectives of this research is thus to explore the level that bullying is considered in practitioners’ practices and in the psychological manual diagnostic tools.

Legally, on the other hand, there is no specific law addressing cyberbullying in the UK, despite the pressure to do so. However, there are legislative provisions that are applicable to both forms of bullying (see review by [39]). Section 49 of the Telecommunications Act 1984 is relevant when a message is “offensive … indecent, obscene or menacing” and intends to cause “annoyance, inconvenience or needless anxiety to another”, whereas Section 5 of the Public Order Act 1986 focuses on behaviour which is “threatening, abusive or insulting”. In 2001, the Malicious Communications Act 1988 was amended to include electronic forms of communication, and therefore captures cyberbullying. If on two or more occasions the conduct of the cyber bully amounts to the harassment of another, the Protection from Harassment Act 1997 may be applicable. If a public electronic communications network is used to send a message that is grossly offensive, indecent, obscene, or menacing an offence will be committed by virtue of Section 127 of the Communications Act 2006. Finally, the Defamation Act 2013 is relevant where a message has “caused or is likely to cause serious harm to reputation”. If the bullying is racially or religiously motivated, prosecution can be brought under the Crime and Disorder Act 1998 and the Criminal Justice Act 2003 [40]. The responsibility of schools to prevent bullying is set out within the School Standards and Framework Act 1998 and the Education and Inspections Act 2006 [41]. Further research is needed to evaluate if legislation is effective in preventing bullying, and consideration ought to be given to whether specific bullying legislation is a realistic and beneficial measure.

In this study, a qualitative design was chosen to allow for an in-depth exploration of both traditional and cyber forms of bullying, with expert participants who may have to deal with the consequences in their daily work. We sought to examine: how do practitioner psychologists and legal professionals define bullying? How is it perceived in their unique practice? What impact do they think it has on children and adolescents? And finally, what solutions do they recommend?

## 2. Materials and Methods

### 2.1. Participants and Ethical Issues

Purposive sampling and convenience sampling techniques were used in this study to select the participants. In purposive sampling the researcher selects a sample based on their knowledge about the study and population. Participants are selected according to the purposes and needs of the study. The participants were told that the study is expanding on the current literature on bullying by exploring whether it is included within mental health assessments and how the law can be used in instances of bullying/cyberbullying. Lawyers were told that their interviews will be based on bullying and the law, while practitioner psychologists were told that theirs will be based on bullying and mental health. The inclusion criteria were that the practitioner psychologists has dealt with children and/or adolescents as part of their work as psychologists or psychiatrists. Furthermore, participants should have knowledge about bullying, or have worked and dealt with cases who were affected by traditional and/or cyber bullying. These criteria were mentioned in the information sheet and the consent forms, and participants consented to participate in the study based on this.

A total of nine participants (lawyers and psychologists; six males and three females) agreed to take part and completed interviews with no withdrawals from the study (see Table 1). The participants were recruited via internet searches from within or nearby London. In total, 70 lawyers and 94 practitioner psychologists were contacted via email or letter inviting them to participate in the study and explaining the inclusion criteria. Four lawyers (three males and one female) and five practitioner psychologists (three males and two females—three clinical psychologists, one clinical neuropsychologist, and one child and adolescent psychiatrist) met the inclusion criteria. The participants were experts in their field and accurately portray the opinions of others who may also work with people affected by bullying and cyberbullying. Participants volunteered to participate without any compensation. All of the participants were given a written consent form to read and sign prior to the interview, which also included consent to being tape-recorded. In addition, verbal consent was obtained from all of the participants before the interviews were conducted. There were no further ethical issues within the data collection process as participants were not selected based on gender, age, ethnicity, or personal experience of bullying. No questions regarding their personal experience of bullying were asked during the interview. At the transcription stage, some information was not included, such as any personal experiences that were disclosed, detailed information about clients that did not relate to the topic and any identifiable information about themselves or others. In addition, participants were given pseudonyms to ensure confidentiality and anonymity. All of the original tape recordings were destroyed on the completion of the entire study. Ethical approval was obtained from a University ethics committee in the UK prior to data collection.

### 2.2. Interviews and Data Collection

Interviews were conducted at the participant’s place of work or home at a time and date that was convenient for the participants. They lasted between 25 and 55 min. The interviews were recorded using an Olympus WS-812 digital voice recorder. An interview protocol was devised based on the existing literature and the help of experts in the fields of psychology and law (see Supplementary Table S1). The protocol was differentiated for lawyers and practitioner psychologists, although there was some overlap between the questions. Due to the semi-structured nature of the study, additional questions were asked of each participant based on their responses to previous questions. This structure allowed for a full exploration of what the topic meant to participants in terms of their professional expertise. The interview protocol was pilot tested on two people.

### 2.3. Data Analysis

The transcription process was conducted following each interview and the recordings were transcribed verbatim by one of the researchers. Thematic analysis was used because it offers a flexible data analysis approach. The six phases of thematic analysis were implemented, as outlined by Braun and Clarke [42], and were conducted separately for the lawyers and practitioner psychologists. The first three stages of analysis were conducted separately for each transcript to ensure that the maximum range of data was gathered. The first step was familiarisation with the data. Each verbatim transcription was read a minimum of two times and then preliminary notes were made on the second reading. The second stage of thematic analysis was initial coding, based on relevant data to the research and were recorded in the right hand column of each transcript. The third stage was searching for themes, identifying further codes and drawing together each transcript; repetitive codes were recorded on a mind map. An analysis of each of the codes was completed at this stage and how different codes worked together. For each potential theme all of the data extracts were collated together in a CodeBook. The fourth stage involved reviewing the themes, with re-reading of the transcripts; the main themes began to be identified and it was ensured each theme was holistic and independent from the others. The fifth stage involved defining and naming each theme and checking them against the research questions. The sixth stage was writing up the themes and selecting data extracts to represent the theme appropriately, again reviewing the transcripts and CodeBook. Two coders independently constructed the themes that were then jointly reviewed and decided with the help of a senior reviewer. Cohen’s kappa was computed for the constructs and the results revealed very good inter-rater agreements; all kappa’s exceeded 0.87. All of the discrepancies were discussed and resolved by the coders.

## 3. Results

The thematic analysis across both groups of participants revealed three main themes regarding both traditional and cyber bullying: (1) What does it entail? (2) Current professional procedures; and (3) Education is key.

### 3.1. Theme 1: What Is Bullying?

Overall, the practitioners and lawyers were familiar with the concepts of traditional and cyberbullying, and had a range of professional experience of such incidents with young people and adults. As such, the first and shared theme for all of the participants provided a platform for them to discuss what they believed to be the main facets and implications of bullying when drawing from their own experiences.

#### 3.1.1. Definition and Characteristics

Overall, all participants identified at least one of the key elements of the standard definition of bullying, including the unkind nature, the power imbalance, intentionality, and repetition. However, their unique definitions were not entirely holistic and in general their individual answers did not account for all of the facets of the bullying definition:

*“Telling the wider world something unpleasant about someone but there’s also the frequency and intensity of the one to one interaction”*.

(T.F., Practitioner Psychologist (PP))

Cyberbullying was believed to be similar to traditional bullying, but using different tools, such as the Internet and electronic devices, to target victims. However, the general speculation was that the characteristics of cyberbullying led to the consequences being more devastating than for traditional bullying. Various reasons were given for this belief, including the issue of anonymity and physical distance created by using the Internet to communicate:

*“… I think people can hide behind anonymity and that they are protected from disclosure of identity which isn’t actually the case. I think that gives a false confidence but also I think it dulls the emotions of the attacker because when you are, you know, in the playground bullying for example, you are having to face your victim face to face so you actually see their reaction. Whereas if it is online or via a mobile you can’t see their reaction so your senses are essentially dulled.”*.

(L.G., Lawyer (L))

#### 3.1.2. Impact

The psychological impact bullying has on the victim’s life was stressed throughout all of the interviews. In general, the practitioner psychologists felt that bullying significantly contributed towards psychopathology and directly influenced the psychological health of a victim. Despite the lack of professional experience in this area, the lawyers shared similar views and mentioned several negative consequences (e.g., suicide) of bullying. The range of psychological impacts that were mentioned were vast and accounted for less severe consequences, such as poorer school performance and lower self-esteem, to more severe clinical diagnoses, including depression, anxiety, post-traumatic stress disorder, and eating disorders:

*“Self-doubt, reduced self-esteem, depression and anxiety, lack of trust in the world. Probably is some reduction in self-efficacy, probably some sort of externalising …it can also lead to anger, aggression, in the right circumstances a paranoid ideation”*.

(S.H, PP)

There was a tendency for all of the participants to concentrate on the outcomes for the victims and it was only when prompted that they discussed the other groups, suggesting there is less knowledge or awareness about the impact on bullies, bystanders, and bully-victims. In general, it was felt that the psychological impact was less severe for the bully and that it was difficult to determine what outcome it might have on their mental health. Responses were mixed and less certain regarding the long-term or psychological effects (if any at all) for the bully. It was felt that this depends on the bullies themselves and their reasons for engaging in the behaviour. One suggestion was that bullying could be used as an avoidance strategy where they were getting a strong sense of control that brings some relief from their own pain. Guilt was one emotion that was speculated to be strong for bystanders, particularly in the future when they look back on the event and feel shame for not stopping it.

*“have some negative consequences for themselves in terms of I’m a coward, why didn’t I do something”*.

(S.H., PP)

### 3.2. Theme 2: Current Professional Procedures

For the practitioner psychologists, current procedures mainly referred to how bullying was considered in psychological assessments and interventions. In contrast, the lawyers focused on current laws and who was currently and legally responsible for preventing or intervening in such cases. These strands are outlined separately below.

#### 3.2.1. Practitioners’ Professional Procedures

The practitioners displayed a mixed usage of diagnostic manuals in their practice. Some argued that these manuals did not inform professionals outside of their own knowledge base. Instead, they used their own professional training and the latest research to guide their clinical work:

*“If I had some stats that said you know, which I’m sure there are for children, but you know X percentage of the general population suffers some form of bullying at a certain point that would give it more legitimacy”*.

(S.H., PP)

Indeed, some of the practitioner psychologists believed that including bullying within diagnostic manuals may be beneficial or something that they could consider during their own assessments. Although they believed this to be a worthwhile endeavour, they all admitted that they do not currently include such questions in their assessments. Instead, they wait for the client to raise this issue themselves. Only when the client indicates that they have been involved in a bullying event will they explore this issue further. In general, this was because the assessment period was believed to be too restricted to include extra assessments:

*“in assessment the difficulty is you’ve got about a million other things that also have to be assessed at the same time so a lot of it’s informed by what the person is bringing to you”*.

(A.S., PP)

These practitioners believed that engaging the health services is essential in terms of intervention strategies for both bullies and victims. One practitioner noted that the individuals who have asked for help and had not received it are a particularly high-risk group. This issue of immediate intervention was raised frequently throughout the interviews as this can help prevent adult psychopathology:

*“If it was managed at the time then you don’t have anything to manage later”*.

(A.S., PP)

It was suggested that more of an effort should be made to encourage and ensure an open dialogue about bullying in all schools and workplaces so that victims or bystanders do not feel shame when reporting it. Indeed, being able to openly discuss such an event at the time could allow for a faster healing process for the victim:

*“a huge thing that makes a difference from my clinical experience is having somebody around you that you could speak to at the time”*.

(A.S., PP)

Furthermore, the practitioner psychologists also identified that there is a need for a legal element to bullying and for standard consequences for those who engage in it. Mainly, they felt that the psychological impact can be so severe on the victims that legalisation is necessary. Indeed, they all agreed that they would take legal action if they were presented with a case where they felt it was warranted:

*“there will probably be a need for some kind of legal point for those who are the extreme offenders”*.

(T.F., PP)

#### 3.2.2. Lawyers’ Professional Procedures

Although there is no specific and unique law for bullying and cyberbullying, the lawyers mentioned several existing laws that can be utilised in such cases, including the Equality Act 2010, the Malicious Communications Act 1988, Protection from Harassment Act 1997, and the Public Order Act 1986. These statutes can be utilised in different ways depending on each case and each victim. However, their effectiveness for dealing with bullying cases is debatable and questions were raised as to whether they were actually implemented as much as they could be. This point was consistently raised in terms of cyberbullying and there was a consensus that these laws are limited in combating this type and relatively new form of behaviour-one that has emerged since these statutes were enacted:

*“with the scale of cyberbullying it’s actually quite difficult to tackle”*.

(L.G., L)

In terms of moving this area forward, there were mixed views on whether a new and specific legislation could and should be created that would consider the unique legalities of bullying and cyberbullying. One lawyer felt that this was a necessary and worthwhile step, particularly for cyberbullying. However, the majority were more cautious and felt that there was still a lot of work to be done in the area of cyberbullying in particular, and that having one piece of legislation for these negative behaviours might be a little overly simplistic:

*“there is work to be done. Er but that doesn’t mean to say you rush into something too quickly just because you know where you are at the moment isn’t very good […] if you rush into something which is equally a bit of a mess then that is not really an improvement either”*.

(C.H., L)

For the most part, it was believed that efforts should be focused on the implementation of the current legislative provisions, something that has not been done to date. Indeed, the perceived ineffectiveness of these laws might be as a result of them not being enforced sufficiently. The lawyers highlighted several other problems with using existing laws to deal with cyberbullying cases in particular. The most problematic was the lack of expertise within the police system and police resources. Police officers are not often experts in technology, and training in online monitoring might not be prominent in their professional history. As such, intervening in online events may not be as prevalent as in offline environments:

*“the officers […] they’re not specialists in computers and patrolling online, that’s not within their remit”*.

(P.K., L)

A second problem with existing legislation is the associated financial costs, which the lawyers believed prevents bullying cases reaching the courts. Indeed, there are costs that are associated with cyberbullying that might not be there for traditional, such as the process of identifying people who were initially anonymous. Overall, bringing a bully to court was stated to be a costly endeavour and one that is not affordable for all victims:

*“it’s very disappointing for me as a lawyer when we have people ringing up all day saying I’m being cyberbullied and being stalked but they can’t afford you know to write a letter to try and deal with it. Erm and I think the system lets victims down on that regard”*.

(P.K., L)

The last problem focused on the jurisdictional issues that were noted as a potential to prevent the law being used effectively. It is very difficult to legally intervene with cyberbullying when the bullying is taking place in another country, which may have its own legislation for such events. As such, because of the geographical distance between the victim and perpetrator, the victims are only partly protected by the law:

*“if bullying is being done from abroad it would be very difficult to stop it from happening and to prosecute”*.

(J.L., L)

### 3.3. Theme 3: Education Is Key

The most imperative prevention strategy for bullying identified by the practitioner psychologists and the lawyers alike was education. Education is considered as vital for bullies to take the perspective of their victims and to consider the consequences of their actions. In addition, victims would benefit from education in terms of their coping strategies so that they may increase their resilience to negative experiences.

It was consistently believed that increased education and awareness would reduce prevalence rates of bullying amongst all ages. In particular, it was argued that bullies or cyberbullies might not understand their behaviour because of a lack of awareness and education about what bullying entails, particularly when it comes to children and adolescents. As a consequence, they might not perceive themselves to be perpetrators, and as such, cannot be expected to understand the seriousness of their behaviours:

*“I see adults who were bullies and didn’t realise they were bullies at the time and reflect later that they were actually bullies and they didn’t understand that was what they were doing”*.

(A.S., PP)

Education needs to be directed towards the general population as a whole and not just young people or schools. This will ensure that everyone is knowledgeable about the definition of bullying and its consequences, who is responsible for combating it and what the best strategies for stopping it are. This education could be generated in a range of different ways, including skills training for the parents of victims and bullies; legal training for employers and schools; technology training for the police; and individual coping strategies for young people and adults who have direct experiences of bullying:

*“education for young people [should be] about safe internet use so they know how to recognise bullying if it happens for them and where they can go for help and the importance of speaking up and not trying to manage it on your own”*.

(R.Q., PP)

## 4. Discussion

This study took bullying research a step forward by investigating debates in the current literature with professionals who know the implications of such experiences. The study is the first of its kind and can form the basis for future quantitative and qualitative studies with bigger and more comprehensive sample sizes. This is to verify the conclusions of this study about the necessity of performing revisions in the legal policies and clinical psychological practices regarding traditional and cyber bullying.

Bullying is a prominent societal phenomenon that the majority of people will experience at some point in their life, not just in childhood. As technology develops rapidly over short periods of time, younger generations will undoubtedly become more technologically advanced. The practitioner psychologists described this as a developmentally challenging time, supporting the idea that bullying can be traumatic [31] and a serious health risk [43]. Both practitioner psychologists and lawyers (despite it not being the area of expertise for the latter) were aware of the mental health consequences that are associated with bullying. They also supported the recommendation that education and communication are the best interventions [44], with legal interventions deemed necessary for the serious cases where an individual’s mental health is in jeopardy [45,46]. Of course, individual differences need to be considered for legal cases and not everyone will develop a psychological disorder as a result of being involved in bullying and/or cyberbullying.

### 4.1. Definition and Interventions for Bullying and Cyberbullying

Practitioner psychologists and lawyers felt that education and communication are vital intervention strategies and should not just be targeted towards children. The practitioner psychologists believed that education ought to include the definition and the consequences of bullying and cyberbullying and the subgroups involved (bullies, victims, bully-victims, and bystanders). Whilst the lawyers felt that education should consist of information about how identities of anonymous users can be disclosed [5,47], there is an element of permanency about the internet [45,48] and the benefits of technology [49]. Education is important to highlight, because bullies are not always entirely knowledgeable about the consequences of their actions [40] and may not perceive themselves to be bullies. However, practitioner psychologists realistically understood that not everyone will be receptive to education, although they believed that the majority of people would be helped through greater awareness about bullying. In addition, raising awareness that bullying is a criminal offence may be a stronger deterrent than education alone [5,45,46,50,51,52].

It is imperative that there is education about appropriate online behaviour because “effective law tends to reinforce, rather than in itself change, social attitudes” [53]. Thus, a good moral compass needs to be taught at a young age before children start to use any form of technology. Overall, everyone (from website creators, parents, schools, employers, community groups, police, practitioners, and children) should have a basic conceptualisation of what bullying is and the consequences it can have both legally and psychologically.

It is crucial that such education starts early and is brought in rapidly, reinforced by the lawyers’ belief that there is no need to wait for the phenomena to get any worse than it currently is. It is also important that schools include cyberbullying within their anti-bullying policies, as this is often insufficiently covered [54]. It was implied by a practitioner psychologist that bullying and cyberbullying are not completely preventable, which may very well be the case; however, a lot can be done to prevent the majority of bullying happening. It may be that a minority of perpetrators will be recalcitrant to ordinary educational and policy work in schools [55], however this is when the work of the law and psychology combined will be vital.

The practitioner psychologists believed in the importance of immediately applying interventions to prevent negative consequences in adulthood, which is in support of Williams and Godfrey [32]. Furthermore, lawyers clarified in terms of the law that if bullying is reported, liability arises for those who the incident is reported to. However, previous research suggests that victims of bullying are unlikely to discuss their experience [47,50,56,57,58,59], which can also jeopardise their mental health [60]. In addition, victims perceived adults to be technologically incompetent [2,44], thus being unable to offer help and support. This further supports the lawyers’ belief that parents lack knowledge about online behaviour and lack control over their children’s internet or technology use.

The internet has been described as lacking restrictions [50,61] along with a lack of parental supervision [62]. However, parental supervision is considered a vital component for bullying intervention [46,56,59,63], thus needs to be implemented. In addition, online supervision could potentially ensure internet or technology use is monitored without impinging on the rights of free speech and should ensure long-term positive effects as children grow up to use technology as adults. However, control and monitoring (e.g., investigating which websites has been visited) are not always effective in reducing cyber aggression (e.g., [64]). Furthermore, there is a generation gap in terms of technological knowledge and skills between adolescents and parents on the one hand [65], and adolescents and mental health practitioners on the other hand. In some instances, parents may not be fully aware about online risks and believe that their children are able to recognize them and thus they reduce the level of monitoring [66].

Online and digital devices are particularly hard for parents to monitor for several reasons. They are technologically complex; market innovation requires parents to continuously adapt and update their habits, and at times parents might feel that they are outsmarted by their children who are often more skilled than them. Digital devices are increasingly more personalised and portable, and as a result, traditional methods of monitoring are becoming less effective [67,68]. This could lead to increased online problems. Parents and mental health practitioners are, therefore, strongly encouraged to take active interest in adolescent ICT activities [66] and invest in understanding the mechanisms and consequences of new technologies.

Consequently, a collective responsibility and proactive stance should be taken by a range of people; legal personnel, internet sites, parents, and practitioner psychologists, to help to prevent and protect those that are involved in bullying and/or cyberbullying. Education and communication could be the first steps in reducing the prevalence of these behaviours. These interventions help alleviate some of the psychological harm and ensure bullying is dealt with swiftly and effectively.

The lawyers felt that there is considerable behavioural change when using the Internet [69] due to anonymity and physical distance [17,70], which has been found to result in an online disinhibition effect [71]. However, there was consensus that offline and online behaviour should be treated the same in terms of criminality and appropriateness, which also needs to be communicated to the wider public.

Collectively, the participants were able to cover all of the aspects of the bullying definition (repeated, intentional, and include power imbalance), although individually their definition lacked specifics. Of course, being asked to describe any commonly used word is difficult, however, it appeared it was more than just a lack of recalling an accurate definition. The practitioner psychologists identified that the definition of bullying and cyberbullying is down to individual perception. This creates problems for understanding and dealing with such incidents. For example, a victim needs to be confident that their experience is defined as bullying in order to disclose it to another person and go through court proceedings if necessary. In order to use the law to its full advantage, specific elements of a behaviour need to be present. To use the law, bullying is described as being grossly offensive, indecent, threatening, abusive, and insulting, which causes distress, anxiety, and annoyance to the recipient (victim). Yet, these words are not encompassed within the current definition of bullying and cyberbullying, thus raising the question of whether a more cohesive definition needs to be created. A more accurate, holistic, and commonly known definition, which includes these legal aspects, would provide simplicity and clarity. It will be of advantage particularly when assessing whether legal action is appropriate.

### 4.2. The Psychological Risks of Bullying and Cyberbullying

In accordance with previous quantitative research [12,13,31], the practitioner psychologists identified a broad range of disorders that are associated with being a victim, including post-traumatic stress disorder, depression, anxiety, eating disorders, and substance misuse. Furthermore, other psychological consequences were raised, beyond the findings of previous research, which includes obsessive-compulsive disorder, body dysmorphic disorder, and a negative view of the world. The practitioner psychologists believed a negative view of the world to be a detrimental and long-term consequence for victims because it can affect different areas of their lives, such as trust and confidence in relationships. Previous research found that victims can experience long term problems in relation to their health, finances, and socialisation [14,15]. In addition, they felt that the shame that victims experience as a result of being bullied results in reduced disclosure and more internal struggling, which contributes to psychological distress. Each practitioner psychologist, based on their expertise, identified at least one psychological disorder and believed bullying to be prominent in the majority of their client’s histories. This implies that their understanding about psychological disorders of bullying and/or cyberbullying is based purely on clinical work with clients. Furthermore, the practitioner psychologists believed that bystanders and perpetrators mainly experience guilt. There was also little recognition of the psychological consequences for bully-victims despite this group being consistently identified as the most vulnerable to psychological distress [13,17]. This confirms there is not enough awareness amongst practitioner psychologists regarding the psychological impact on others besides victims. This may also imply that there is not adequate support available for these high-risk individuals. It is important to stress that bullying and cyber bullying research is substantial, presenting wide spread knowledge to us all, but although there is consistency across studies and research, there are also discrepancies. Such discrepancies are often hard to moderate and assess presenting potentially conflicting and challenging content.

### 4.3. Bullying and Cyberbullying within Psychological Assessments

Previous research has called for questions relating to bullying to be included within psychological assessments [17,72]. Despite the practitioner psychologists believing in the importance of discussing bullying, they waited for the clients to raise and discuss such experiences. This reinforces the idea that there is still “a huge void between knowledge of the adverse consequences of bullying and awareness, enquiry and intervention by healthcare providers” [33], and highlights the gap between research and practice. It is possible that people are more likely to discuss bullying experiences in a therapeutic setting. However, those who do not raise it may be struggling with the impact of the experience and consequently will not receive support from professionals. Preventative measures need to be in place to reduce the psychological impact earlier and interventions need to be implemented at the time that the bullying is being experienced.

The practitioner psychologists noted that they should be more proactively addressing cyberbullying within their client work. However, time was the main reason for not asking specifically about bullying or cyberbullying in assessments. Diagnostic manuals, such as the DSM-5 [37] and ICD-10 [38], were considered secondary to previous training and research in regards to how they approach their clinical work. Therefore, it can be argued that bullying needs to be incorporated into training programmes, which is also suggested within NICE guideline CG28. This will increase the awareness of bullying and encourage professionals to consider the latest research on bullying in their sessions with clients. Practitioner psychologists would also be invaluable when a victim is going through court proceedings as it could be a psychologically difficult time [73].

### 4.4. The Legalities of Bullying and Cyberbullying

Both the practitioner psychologists and lawyers agreed that a legal intervention is vital for bullying because of the risks of negative outcomes (e.g., suicides). To understand the law that is applicable to bullying, it was suggested that speaking with “relevant … legal counsel” [48] is a priority, which this study achieved.

Bullying can be considered illegal under a number of legislative provisions (Legislation.gov.uk), namely; the Telecommunications Act 1984, Public Order Act 1986, Malicious Communications Act 1988, Protection from Harassment Act 1997, School Standards and Framework Act 1998, Communications Act 2006, Education and Inspections Act 2006, the Equality Act 2010, and the Defamation Act 2013. The lawyers believed the Equality Act 2010, Malicious Communications Act 1988, the Protection from Harassment Act 1997, and the Public Order Act 1986 were the main Acts that could be used to deal with bullying cases. Despite bullying being illegal, lawyers did not believe that there is sufficient awareness of this amongst the general public. Increased awareness could be achieved via specific legislation and education. However, the lawyers did suggest that there are sufficient existing provisions available, and thus did not feel that a specific Act would be appropriate at present.

During the completion of this study, the House of Lords created a Committee to review whether a specific Act was needed. It was decided the existing legislation is “generally appropriate for the prosecution of offences” [53], which supports the view of the lawyers in this study. However, there is some speculation that the current legislation is not entirely effective [52]. The main reason for this is that the legislation was created when cyberbullying was not a societal problem and when technology was not an everyday necessity. However, the lawyers in our study felt that once the legislation is reviewed and enforced there would be more successful prosecutions. Under UK legislation there is not a specific law that clearly makes cyberbullying illegal, although it can be considered a criminal offence under different legislation. For example, under the Protection from Harassment Act 1997, it is a criminal offence for a person to pursue a course of conduct that amounts to the harassment of another, which the perpetrator knows or ought to know amounts to harassment. This could include sending a person multiple abusive emails with the intention of causing alarm or distress. Section 1 of the Malicious Communications Act 1988 states that it is an offence for any person to send a communication that is “indecent or grossly offensive” for the purpose of causing “distress or anxiety to the recipient”. The Act also extends to threats and information that is false and known or believed to be false by the sender of the communication. Section 127 of the Communications Act 2003 makes it a criminal offence to send via any electronic communication network a message or other matter that is deemed “grossly offences or of an indecent, obscene or menacing character”. The Obscene Publications Act 1959 makes it an offence to publish an obscene article. An obscene article is classed as one whose effect is to deprave and corrupt persons likely to read, see, or hear the matter contained or embodied in the article. Publishing includes circulating, showing, playing, or projecting the article, or transmitting the data. Under Section 5 of the Public Order Act 1986, it is an offence to use threatening, abusive or insulting words, behaviour, writing, or any visual representations that is likely to cause harassment, alarm, or distress within the hearing or sight of a person. With regards to cyberbullying, this offence could apply where the camera or video functionality now found on the vast majority of mobile phones is used as a way of causing such harassment, alarm, or distress. If in the course of cyberbullying a person hacks into the victim’s online accounts or personal computer, they may be committing an offence under the Computer Misuse 1990 (for more information see: [39]). In the United States (US) on the other hand, as of December 2017, forty-nine states (all but Montana) have authorised bullying prevention laws (for a regularly updated list of state legislation see: www.laws.cyberbullying.org). All of these require schools to have policies to deal with bullying, and almost all of them refer to criminal sanction for cyberbullying or electronic forms of harassment (44 states) or specific school sanction for cyberbullying (45 states), but there exists great variation across states regarding what exactly is mandated. In Qatar, recently, there have been some positive initiatives, such as a Cybercrime Prevention Law, the development of a National ICT Strategy, and a website detailing safe practice guidelines for Internet usage [74].

Other problems besides legislation exist, such as police resources, financial costs, and jurisdiction problems. The lawyers felt that these problems needed to be tackled separately. This reinforced the idea that a specific Act would not be effective at this time because it would not overcome these problems. Two important changes that are currently being considered are using private prosecution to eradicate the financial costs and specialist police units to ensure that reports of bullying are investigated sufficiently. However, it is hard to overcome the jurisdictional problems, thus the law can only protect victims to a certain extent. On the whole, the suggestions from previous research that specific legislation is the main solution to the legal problems appears unrealistic. Instead, efforts should be focused on improving how incidents of cyberbullying are dealt with by the police and the prosecution process to ensure that a more efficient legal intervention is achieved.

Moreover, the Internet provides both opportunities and risks. According to UNICEF [75], the Internet has positive impact on the lives of young people, allowing them to develop their digital capacities and increase their opportunities for learning. Despite the positive effects, internet and ICT, in general, pose challenges often translated in online safety. Cyberbullying should not be looked at in isolation from other online risks. Despite internet and digital devices offering young people opportunities, they also increase the likelihood of experiencing different forms of risks. Livingstone and Haddon [76] reported various evolving online risks that translated into content, contact, and conduct. Content refers to young people receiving online harmful content (e.g., hateful, spams, pornographic), while contact refers to experiencing through the contact with others (e.g., being bullied, meeting strangers, online grooming). Conduct refers to perpetrating or conducting behaviour that pose risks such as gambling, hacking, and bullying others. When combined with the evolution of online communication and the nature of messages (text, images, video and audio) such risks are extremely difficult to control. Online safety and digital literacy are crucial, cyber bullying could intersect with other online risks, and as a result, prevention methods, including potential laws, should be inclusive of all and continuously evolving meeting the digital divide between children, parents, educators and practitioners.

## 5. Conclusions

Overall, this study indicated that interventions need to be adapted and implemented immediately to tackle bullying. This study investigated debates in the current literature about bullying with professionals who are experts on the implications of such experiences. The study can be the first step for a bigger study that can include more practitioners. It would have been also beneficial to interview members of the police force, school personnel, and parents about their current understanding of bullying regarding psychological issues and the law.

There are some limitations for the study. Firstly, the sample size of the study included nine participants. Any future study should include a bigger sample size using mixed method designs including quantitative and qualitative analysis to be able to generalise the findings to specific populations (lawyers and psychologists working with children and adolescents on cases related to bullying) and make stronger recommendations to inform policy change. Nevertheless, the nine participants within this study have explored the debates from previous research and how interventions can be applied in the real world, which is invaluable. Also, the study used a qualitative method to extract the results and opinions of the participants. Secondly, the study used purposive sampling for the inclusion of participants. It is true that there is no intended bias in purposive sampling, but due to a lack of random sampling, purposive sampling is sometimes open to selection bias and error. However, we made sure to eliminate selection bias by relying on the inclusion criteria.

This research extends the existing literature by indicating several areas of improvement and it will be essential for future research to investigate the impact that these changes will have on prevalence levels, associated psychopathology, and the effectiveness of legal interventions. The first area of change is governmental resources for police forces to guarantee they have the expertise and means to investigate bullying cases. The second area is to include a question about experience (past or current) of bullying and/or cyberbullying within psychological risk assessments. This will ensure that all clients are given the opportunity to discuss their experience and allow for further exploration of the experience if necessary. The third area is the need for an immediate increase in education and discussions of bullying, including a detailed definition. Whether this is at home or school it will ensure that appropriate action, psychological or legal, is taken as early as possible. The fourth area of change is an increase in liability for website operators to ensure that internet sites cannot be used anonymously. Thus, guaranteeing users to think more critically about their online behaviour.

## Figures and Tables

**Table 1:** Details of participants.

Initials	Profession	Profession Description	Gender
T.F	Practitioner Psychologist	Consultant clinical psychologist	Male
R.Q	Practitioner Psychologist	Clinic director and clinical psychologist	Female
S.H	Practitioner Psychologist	Consultant clinical neuropsychologist	Male
A.S	Practitioner Psychologist	Clinical psychologist	Female
J.W	Practitioner Psychiatrist	Child and adolescent psychiatrist	Male
P.K	Lawyer	Technology, communications and EU procurement law	Male
J.L	Lawyer	Principal lawyer, criminal defence	Male
C.H	Lawyer	Cyber risk management, internet monitoring lawyer	Male
L.G	Lawyer	Social media and cyber crime	Female

## Supplementary Materials

The following are available online at www.mdpi.com/1660-4601/14/12/1449/s1, Table S1: Interview Protocol.
