# Supplementary material for: Bullying and Cyberbullying: Their Legal Status and Use in Psychological Assessment
Source: Int J Environ Res Public Health. 2017 Nov 24;14(12):1449. doi: 10.3390/ijerph14121449 (PMC5750868; doi:10.3390/ijerph14121449)
Supplement: Supplementary file 1 [file ijerph-14-01449-s001.pdf]

International Journal of
Environmental Research and Public Health

MDPI

Article

Bullying and Cyberbullying: Its Legality and Use in Mental Health Assessments

Muthanna Samara [1,*], Vicky Burbidge [1], Aiman El Asam [1], Mairéad Foody [2], Peter K. Smith [3] and Hisham Morsi [4]

Table S1: Interview Protocol *.

Practitioners (Psychologists and Psychiatrists)	
**Topic**	**Questions**
	1. Have you worked with clients who have experienced bullying or cyberbullying before?
Definition of bullying/cyberbullying	2. How do you define bullying? 2A (prompt) How important/necessary is intent, power imbalance and repetition in defining bullying?
	3. How do you define cyberbullying? 3A (prompt) How important/necessary is intent, power imbalance, repetition and anonymity in defining cyberbullying?
	4. Why do you think there has been desire for a dedicated legislation to cyberbullying?
Consequences of bullying/cyberbullying	5. What do you believe the consequences of bullying are on an individual's mental health of the:<br>- Victim?<br>- Bully?<br>- Bully-victim?
	6. What do you believe the consequences of cyberbullying are on an individual's mental health of the:<br>- Victim?<br>- Bully?<br>- Bully-victim?
	7. Why do you believe there to be different consequences for bullying and cyberbullying (if appropriate—based on previous response)?
	8. Which mental disorders do you associate with bullying?
	9. Which mental disorders do you associate with cyberbullying?
Diagnostic Manuals/Tools	10. Which diagnostic manual do you rely on (and why)?
	11. Do you think bullying is sufficiently represented in the diagnostic manual and assessment methods (and why)?
	12. Do you think cyberbullying is sufficiently represented in the diagnostic manual and assessment methods (and why)?
Inclusion of bullying/cyberbullying in assessment	13. Do you include experiences of bullying within your assessments of clients (as bullies, victims or bully/victims)? Yes: in what way? No: why not?
	14. Do you include experiences of cyberbullying within your assessments of clients (as bullies, victims or bully/victims)? Yes: in what way? No: why not?
	15. What questions do you/would you ask clients about bullying?
	16. What questions do you/would you ask clients about cyberbullying?
	17. What role do you think the bystander has in bullying with regards to the victims, bullies and the bully-victims mental health?
	18. What role do you think the bystander has in bullying with regards

		to the victims, bullies and the bully-victims mental health?
Legality and responsibility of bullying/cyberbullying	19.	If you know that a client has experienced bullying how would you use this information (e.g., would you take a legal stance and contact the police)?
	20.	If you know that a client has experienced cyberbullying how would you use this information (e.g., would you take a legal stance and contact the police)?
	21.	Who do you believe should be held responsible for bullying (e.g., the bully, the parents, and the government)? And how should they be penalised?
	22.	Who do you believe should be held responsible for cyberbullying (e.g., the bully, the parents, the government)? And how should they be penalised?
	23.	What are the rights and responsibilities of the parents, children and community with regards to victimisation and bullying?
	24.	What are the rights and responsibilities of the parents, children and community with regards to victimisation and cyberbullying?

Lawyers

Topic		Questions
Definition of bullying/cyberbullying	1.	How do you define bullying? 1A. (prompt) How important/necessary is intent, power imbalance and repetition in defining bullying?
	2.	How do you define cyberbullying? 2A (prompt) How important/necessary is intent, power imbalance, repetition and anonymity in defining cyberbullying?
Consequences of bullying/cyberbullying	3.	What do you believe the consequences of bullying are on the mental health of the:<br>- Victim?<br>- Bully?<br>- Bully/victim?
	4.	What do you believe the consequences of cyberbullying are on the mental health of the:<br>- Victim?<br>- Bully?<br>- Bully/victim?
	5.	Why do you believe there to be different consequences for bullying and cyberbullying (if appropriate—based on previous response)?
Legality of bullying/cyberbullying	6.	How can a victim who has developed mental health problems as a result of being bullied be protected/compensated in the legal system?
	7.	What are the legal consequences for bystanders (reinforce or assistant) of bullying?
	8.	What are the legal consequences for bystanders (reinforce or assistant) of cyberbullying?
	9.	Do you think bullying is a serious social problem and should be illegal (and why)?
	10.	Do you think cyberbullying is a serious social problem and should be illegal (and why)?
	11.	Why do you think there has been desire for a dedicated legislation for bullying?
	12.	Why do you think there has been desire for a dedicated legislation for cyberbullying?
	13.	In your experience, how might individuals be convicted of a crime of bullying? Do you think this will change in the future?

	14. What cases and statutes do you tend to rely upon in bullying cases?
	15. In your experience, how might individuals be convicted of a crime of cyberbullying? Do you think this will change in the future?<br>16. What cases and statues do you tend to rely upon in cyberbullying cases?
	17. Should traditional bullying and cyberbullying have different legal consequences?
	18. Should direct bullying and indirect bullying have different legal consequences?
	19. How effective do you think the current legislation is in addressing cyberbullying (and why)?<br>18b. Do you think it would be possible to have a specific law against cyberbullying?<br>Yes: how would this work and what would the law consist of?<br>No: why do you not think it would be possible?
	20. Do you think cyberbullying as an area of law requires immediate reform or would it be more appropriate to allow the offence to evolve further prior to specific legislative action?
	21. Do you think the age of criminal responsibility of 10 is appropriate for bullying cases (and why?)
	22. Do you think the age of criminal responsibility of 10 is appropriate for cyberbullying cases (and why)?
	23. If the bully is below the age of criminal responsibility should the parent be held responsible (and why?)
	24. Should a school be penalised for not having the correct anti-bullying measures in place (e.g., anti-bullying policies/supervised ICT time)?
	25. Should a school be penalised for not having the correct anti-cyberbullying measures in place (e.g., anti-bullying policies/supervised ICT time)?
	26. Should schools have the right to expel a child for the cyberbullying of a fellow pupil that takes place away from school grounds but was started in school and affects the child in school?
Responsibility of bullying/cyberbullying	27. Who else should be held responsible for bullying (e.g., the parents, the government)? And how should they be penalised or censured?
	28. Who else should be held responsible for cyberbullying (e.g., the parents, the government)? And how should they be penalised or censured?
	29. What are the rights and responsibilities of the parents, children and community with regards to victimisation and bullying according to the law?
	30. What are the rights and responsibilities of the parents, children and community with regards to victimisation and cyberbullying according to the law?

* More questions were asked depending on the participant's response—none of a personal nature and if the participant has experienced any type of bullying. Although some questions have been repeated for bullying and cyberbullying they may not be asked directly as written on the interview protocol, participants may be asked if their answer is the same for bullying and if it varies they will be prompted to explain further.
